# Dental and Craniofacial Manifestation of Axenfeld-Rieger Syndrome: A Case Report

**DOI:** 10.7759/cureus.26442

**Published:** 2022-06-29

**Authors:** Sheetal Badnaware, Vinay Kumar Srivastava, Meenakshi Chandel, Pooja Gupta, Punit Fulzele

**Affiliations:** 1 Pedodontics and Preventive Dentistry, Faculty of Dental Sciences,IMS - Banaras Hindu University, Varanasi, IND; 2 Pedodontics and Preventive Dentistry, Sharad Pawar Dental College, Datta Meghe Institute of Medical Sciences, Sawangi (M), Wardha, IND

**Keywords:** genetic, ocular, glaucoma, craniofacial anomalies, axenfeld-rieger syndrome

## Abstract

Axenfeld-Rieger syndrome (ARS) is an autosomal dominant syndrome with a prevalence estimated at 1:50000 to 1:100000 in newborns. It is mainly characterized by ocular, craniofacial, and dental abnormalities. From the pediatric dentist's point of view, early diagnosis of the syndrome from the ocular, craniofacial, and dental manifestation can prevent further abnormalities and ocular complications such as glaucoma. This case report presents a brief description of ARS with the characteristics of craniofacial and dental findings.

## Introduction

The Axenfeld-Rieger syndrome (ARS) is an autosomal dominant syndrome with a prevalence estimated at 1:50000 to 1:100000 in newborns [1] and is characterized by ocular, craniofacial, dental, and periumbilical abnormalities [2]. The pathogenesis of the syndrome is still unknown. Both Axenfeld-Rieger anomaly and Rieger syndrome are the variable expression of the same gene. Axenfeld anomalies with glaucoma were termed Axenfeld syndrome [3]. This syndrome manifests itself in various ways, including dental features.

## Case presentation

An 11-year-old male had reported to the department of pedodontics and preventive clinic with the chief complaint of pain in the upper left back region of the jaw. The child was born from non-consanguineous parents. There is no family history of ARS in any of the family members. Axenfeld-Rieger syndrome had been diagnosed when the patient was five years old with suspected glaucoma.

**Figure 1:**
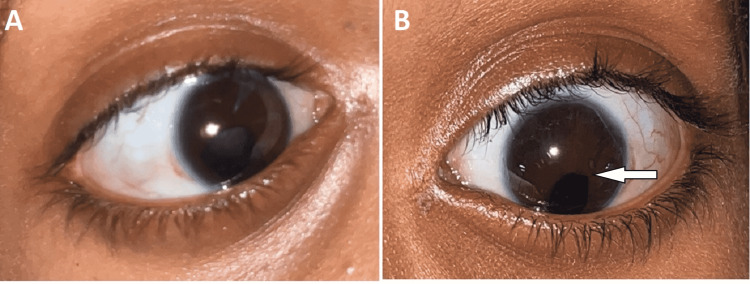
Both eyes of the patient show corectopia

**Figure 2:**
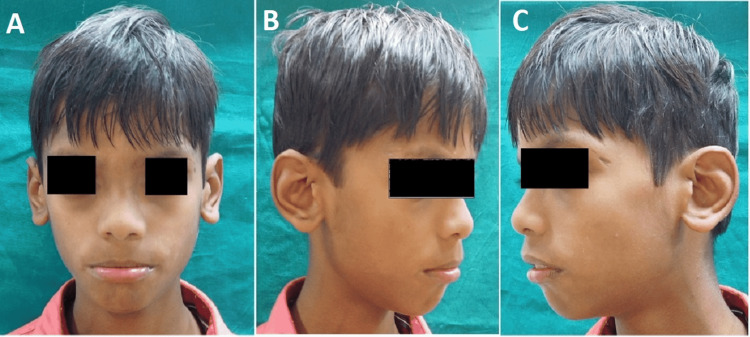
Extraoral features showing depressed nasal bridge, malar hypoplasia, and large pinna size on both sides.

**Figure 3:**
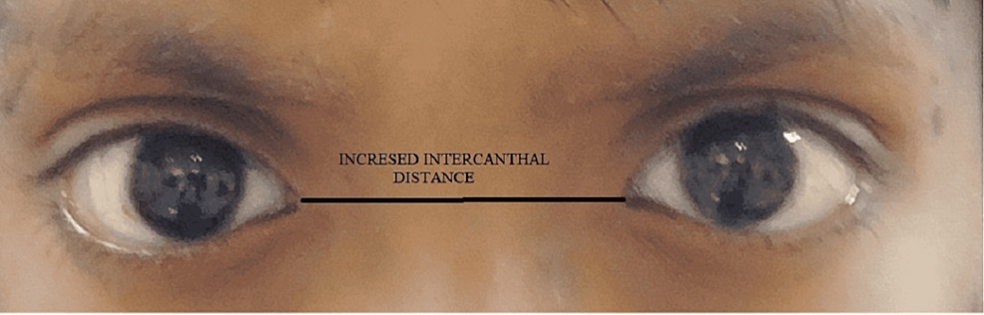
Increased intercanthal distance

**Figure 4:**
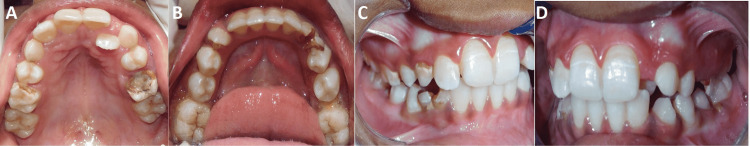
Intraoral examination A: Preoperative view of the maxillary arch, B: Preoperative view of the mandibular arch, C & D: Intraoral view showing class I molar relation on both sides of the arch

A detailed medical examination suggested that the patient has facial and ocular features of ARS, including bilateral megalocornea, type 3 microcornea with iridofundal coloboma, and corectopia with glaucoma suspect (Figure 1). Developmental delay and mental retardation were present. On extraoral examination, the patient had a depressed nasal bridge, malar hypoplasia, and increased intercanthal distance (Figure 2 and Figure 3). The patient showed a symmetrical face with a relatively convex profile and large pinna size on both sides. His lips were incompetent. On intraoral examination (Figure 4), the patient presented with mixed dentition with over-retained deciduous teeth; 53, 55, 63, and 65 were present, and 55 and 65 were grossly carious, and lingually erupting 23 were seen. Occlusal caries was present on 36 and 46 (B of Figure 4). No hypodontia of permanent teeth was observed, and microdontia of permanent mandibular incisor was seen on orthopantomagram (OPG). Root stumps were present for 73, 75, 83, and 84. An Orthodontics evaluation found the class I molar relation on both sides of the arch (C of Figure 4). Soft-tissue examination revealed no other abnormalities except the presence of marked depression of fovea palatini (A of Figure 4).

**Figure 5:**
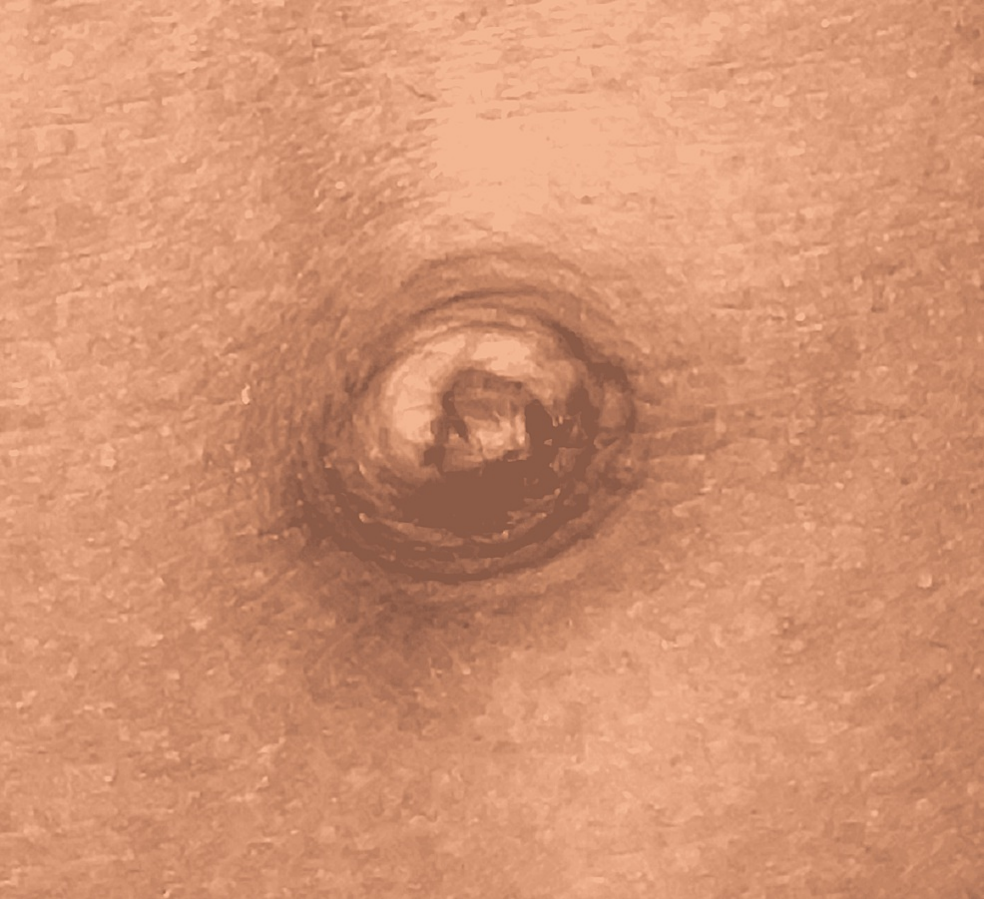
Failure of involution of the periumbilical skin

**Figure 6:**
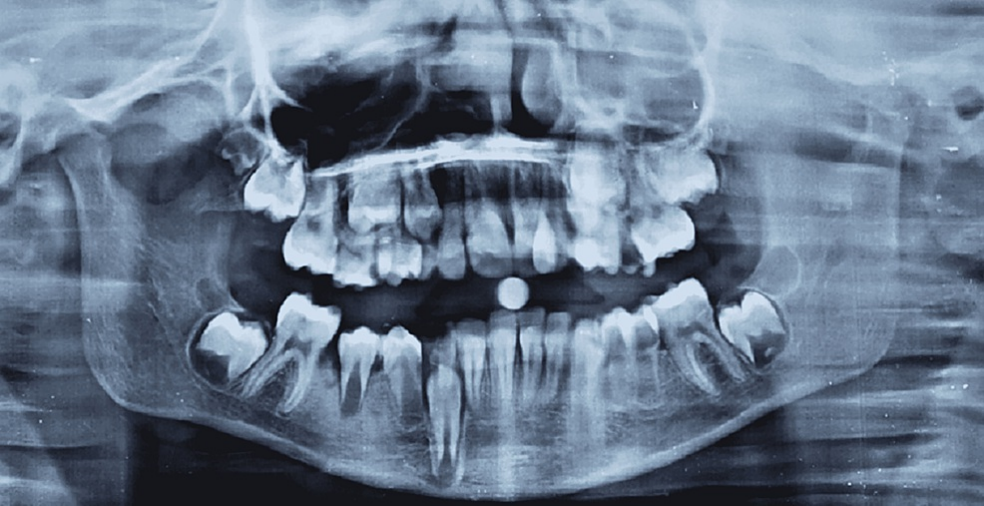
Orthopantomogram (OPG) confirmed the microdontia of permanent mandibular incisor

**Figure 7:**
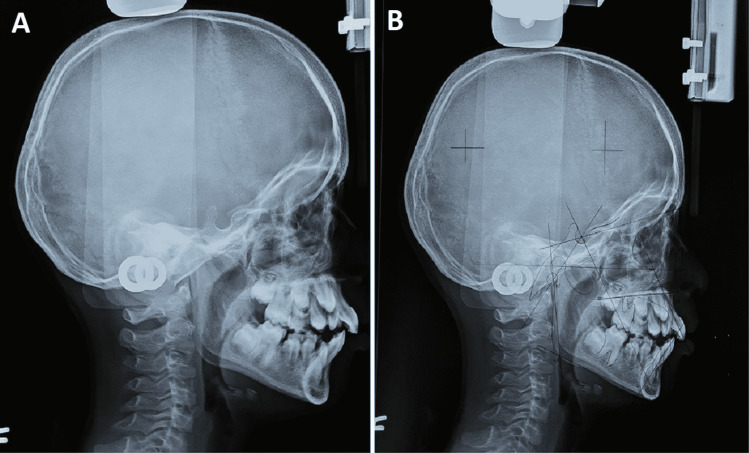
Lateral cephalogram (A) showing relatively large sella turcica, decreased anterior and posterior facial height, hypoplasia of maxilla convex profile. B: Angular and linear cephalometric parameters of the patient

The 2D echo examinations revealed no cardiac abnormalities. On hematological profile examination, only serum alkaline phosphatase level had been increased by 562.3 U/L (normal valve 110-310 U/L); T3, T4, TSH, and growth hormone were within normal ranges. The MRI examinations indicated the presence of hypoxic-ischemic encephalopathy. Audiometry test detected moderately severe bilateral hearing loss with intraocular pressure of 26 mmHg in the right eye and 20 mmHg in the left eye. Also noted was the failure of involution of periumbilical skin (Figure 5). The radiological investigation and OPG confirmed microdontia of the permanent mandibular incisor (Figure 6). The lateral cephalogram was taken with a magnification of 12%. The summary of the tracing and analysis is listed in Table 1. Lateral cephalogram showed an overall decrease in dimensions with few important characteristics of features showing relatively large sella turcica, hypoplasia of maxilla, decreased anterior and posterior facial height, and convex profile (Figure 7). Both the maxillary and mandibular incisors were proclined. 

**Table 1:** Angular and linear cephalometric parameters of the patient SNA angle: The angle between the sella/nasion plane and the nasion/A plane, SNB angle: The angle between the sella/nasion plane and nasion/B plane, ANB angle: Measures the relative position of the maxilla to the mandible, S–N: The distance between sella (S) and nasion (N), PNS-A: The distance between the posterior nasal spine (PNS) and point A

Parameter	Normative Valve	Measurements of the Patient
SNA Angle	82 (deg)	70 (deg)
SNB Angle	80 (deg)	68 (deg)
ANB Angle	02 (deg)	2 (deg)
Frankfort mandibular plane angle (FMA)	25 (deg)	21 (deg)
Y-axis angle	59.5 (deg)	62 (deg)
Anterior cranial base (S-N)	72.71 mm	52 mm
Maxillary base length (PNS-A)	47.78 mm	44 mm

**Figure 8:**
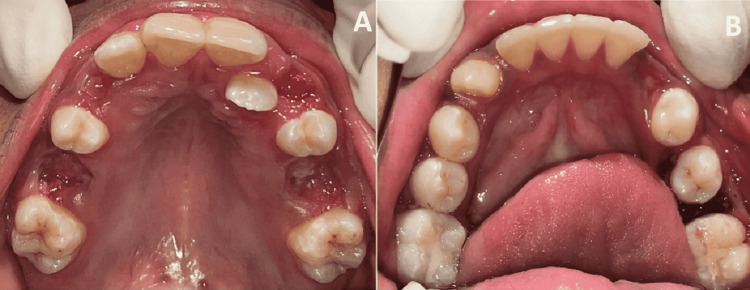
Postoperative view of maxillary (A) and mandibular arch (B)

After obtaining medical consent, the extraction of over-retained deciduous teeth 53, 63, 65, and root stump 73, 75, 83, and 84 were done under local anesthesia using 2% lignocaine with 1:200000 adrenaline (Figure 8). All preventive dental measures were carried out and glass ionomer cement restoration was done with 36 and 46. To ensure proper patient management, a multidisciplinary approach involving an orthodontist and pedodontist were required. 

## Discussion

Axenfeld-Rieger syndrome is a genetic disorder with goniodysgenesis and hypodontia, a craniofacial anomaly with involution of the umbilicus [4]. There are four disorders: Axenfeld anomaly, Rieger anomaly, Rieger syndrome, and Axenfeld syndrome. The ARS refers to a group of disorders that have overlapping conditions of ocular and non-ocular malformations [1]. Axenfled anomaly or Rieger anomaly [5] refers to the condition when only eyes are affected. Rieger described patients with iris hypoplasia, correctopia, and polycoria in 1920, also known as Rieger anomaly. When the Rieger anomaly is associated with glaucoma, it is known as Axenfeld-Rieger syndrome [3]. There have been few descriptions of associated anomalies in the literature, especially dental and craniofacial anomalies that help define the condition.

Mathias et al. first reported dental anomalies in ARS in 1936 [6]. In previous case studies, the involvement of dental and craniofacial anomalies was evident in all patients, as seen in this case report [3,6]. Despite dental anomalies being the most common finding in ARS cases, only very few cases were reported in the literature. Midface deficiency due to alveolar hypoplasia resulting from hypodontia was consistent with findings in other studies by Drum et al. [7] and Jorgensen et al. [8]. No missing permanent teeth are present in this case report; therefore, alveolar hypoplasia may not be related to the number of congenitally missing teeth [9]. Midface hypoplasia associated with ARS is caused due to skeletal and dentoalveolar factors. In the literature, in ARS, class III molar relation is the most common [2,6], but in this case report, there is the presence of class I molar relation on both sides, coinciding with the study by Childers et al. [10]. The height and weight of the patient were within normal limits indicating a normal level of hormones. Lateral cephalogram showing the shape of sella turcica relatively large with both anterior and posterior facial height was decreased. Because of this, most patients with Rieger syndrome are more prone to periodontal breakdown and early exfoliation of teeth. Hence, preventive measures should be undertaken while planning treatment. Glaucoma is seen in 50% of cases of ARS, so consistent intraocular pressure monitoring is required. Although glaucoma can be detected early in infancy, management can be started earlier. The increase in serum alkaline phosphatase level and hypoxemic ischemic encephalopathy has not been reported in the literature, which is present in this case. A marked increase in the serum alkaline phosphatase level can be seen in liver disorder. 

The severity of ARS varies with patients. The management of ARS requires time-dependent treatment and a multidisciplinary approach. The role of a pediatric dentist in diagnosing the ARS cases at an early stage of growth and development could help the patients. Abnormal jaw growth is also a common characteristic feature of ARS. Thus, using functional or myofunctional appliances during the mixed dentition period could reduce the severity of developing occlusion in the adolescent period. In this case, the patient requires a regular ophthalmic appointment to maintain intraocular pressure and optic nerve head changes throughout his life to diagnose glaucoma. Dental treatment aims to preserve the overall health of the oral cavity and aesthetics. Close monitoring of growth and development is essential for such patients.

## Conclusions

Axenfeld-Rieger syndrome is a multisystem disorder with a number of disturbances such as maxillary and mandibular hypoplasia, hypodontia, enamel hypoplasia, and microdontia. The pediatric dentist plays a significant role in the early diagnosis of ARS as most of the findings involve dental, craniofacial anomalies. An early diagnosis could prevent ocular complications such as glaucoma. The treatment plan for this condition involves the preservation of the overall health of the oral cavity and aesthetics. It is imperative for patients with ARS to be closely monitored concerning their growth and development for timely preventive measures. 
